# Supplementary material for: Engineered Expression of Vip3A in Green Tissues as a Feasible Approach for the Control of Insect Pests in Maize
Source: Insects. 2023 Oct 6;14(10):803. doi: 10.3390/insects14100803 (PMC10607264; doi:10.3390/insects14100803)
Supplement: Supplementary file 1 [file insects-14-00803-s001.zip › insects-2602021-supplementary.pdf]

Supplementary Materials

Table S1. Primers used for transgenes identification

Genes	Forward primer	Reverse primer
Zm1rbcS	ACGACGGTGAAACGAAGGAA	GTTCGAACGCTAGAGCCTGT
Cre	ATTTGCCTGCATTACCGGTC	TCAGAAAACGCCTGGCGATC
Vip3A	ATTTGCTTGGTACTGTTTCTTTTGTCGA	GATGCCGTTGAAGTAGTCGATGAA
18S rRNA	CTGAGAAACGGCTACCACA	CCCAAGGTCCAACTACGAG

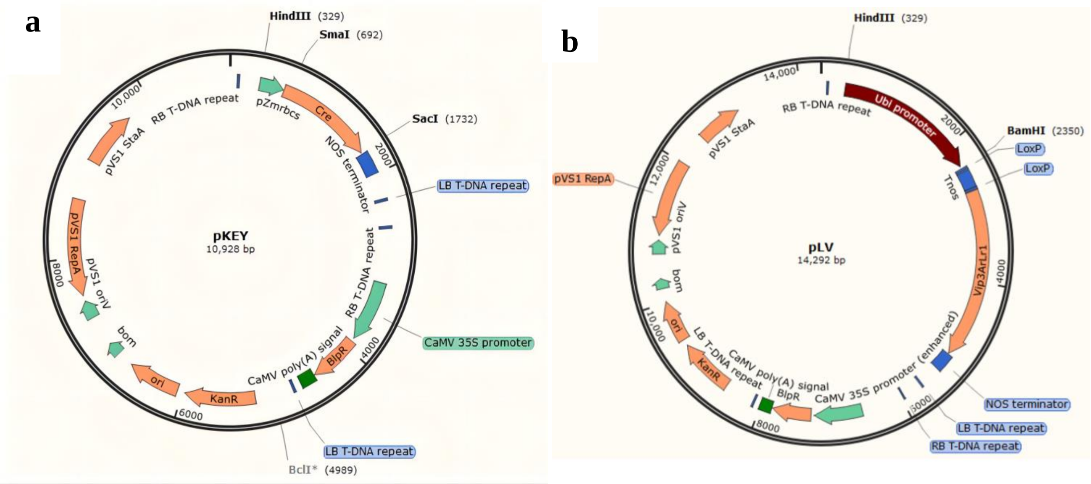

Supplementary Figure S1. Fusion gene cassettes pKEY and pLOCK were constructed in the Cre/loxP-mediated system. (a) The pKEY vector. The green tissue-specific promoter *Zm1rbcS* was cloned in front of the *Cre* recombinase gene tagged with the nuclear localization sequence (NLS) of *Arabidopsis Krp2* to trigger the recombination expressing in green tissues. (b) The pLOCK vector. The pLOCK cassette was constructed with the strong constitutive promoter *ZmUbi* following a *Nos* terminator which was embedded into two *loxP* sites as a lock to block the expression of the *Vip3A* gene. The plant binary vector pCAMBIA3300 was used as basic plasmid. The *hygromycin B phosphotransferase* (*HPT*) gene in pCAMBIA3300 was served as a selectable marker for transformation.

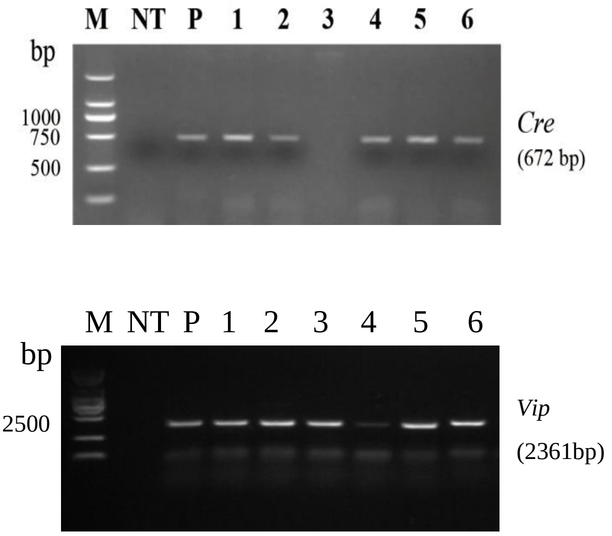

Supplementary Figure S2. Photographs of transgenes with PCR products obtained on T_2 transgenic maize plants. (Above) The *Cre* gene was present in individual KEY transgenic plants. (Below) The *Vip3A* gene was present in individual LOCK transgenic plants. Lane M represents DNA ladder as a size marker. NT indicates non-transgenic plants KN5585. P indicates control plasmid. Lanes 1-6 represent PCR products of individual transgenic T_2 maize plants.
